# TUBB, a robust biomarker with satisfying abilities in diagnosis, prognosis, and immune regulation via a comprehensive pan-cancer analysis

**DOI:** 10.3389/fmolb.2024.1365655

**Published:** 2024-05-02

**Authors:** Zaifu Zhu, Wei Zhang, Shaohu Huo, Tiantuo Huang, Xi Cao, Ying Zhang

**Affiliations:** ^1^ Department of Pediatrics, First Affiliated Hospital of Anhui Medical University, Hefei, Anhui, China; ^2^ Department of Rehabilitation Medicine, First Affiliated Hospital of Anhui Medical University, Hefei, Anhui, China; ^3^ Department of Pharmacy, First Affiliated Hospital of Anhui Medical University, Hefei, Anhui, China; ^4^ The Grade 3 Pharmaceutical Chemistry Laboratory of State Administration of Traditional Chinese Medicine, Hefei, Anhui, China; ^5^ Department of Pathology, First Affiliated Hospital of Anhui Medical University, Hefei, Anhui, China; ^6^ Pathology Center, Anhui Medical University, Hefei, Anhui, China

**Keywords:** TUBB, diagnosis, pan-cancer, prognosis, TME

## Abstract

**Purpose:**

TUBB can encode a beta-tubulin protein. At present, the role of TUBB has not been ascertained in cancers. Hence, the importance of further systematic pan-cancer analyses is stressed to explore its value in the diagnosis, prognosis, and immune function of cancers.

**Methods:**

By collecting and handling integrative data from the TCGA, Firehose, UCSC Xena, cBioPortal, GEO, CPTAC, TIMER2.0, TISCH, CellMiner, GDSC, and CTRP databases, we explored the potential diagnostic and prognostic roles of TUBB in pan-cancers from multiple angles. Moreover, the GSEA analysis was conducted to excavate the biological functions of TUBB in pan-cancers. In addition, survival profiles were described, and the differential expressions of TUBB in different molecular subtypes were discussed. Also, we utilized the cMAP function to search drugs or micro-molecules that have an impact on TUBB expressions.

**Results:**

Based on the TCGA data, we found that TUBB was differentially expressed in a variety of tumors and showed an early-diagnostic value. Mutations, somatic copy number alterations, and DNA methylation would lead to its abnormal expression. TUBB expressions had relations with many clinical features. What’s more, TUBB expressions were validated to be related to many metabolism-related, metastasis-related, and immune-related pathways. High TUBB expressions were proved to have a great impact on the prognosis of various types of cancers and would affect the sensitivity of some drugs. We also demonstrated that the expression of TUBB was significantly correlated to immunoregulator molecules and biomarkers of lymphocyte subpopulation infiltration.

**Conclusion:**

TUBB and its regulatory genes were systemically analyzed in this study, showing that TUBB had satisfying performances in disease diagnosing and prognosis predicting of multiple cancers. It could remodel the tumor microenvironment and play an integral role in guiding cancer therapies and forecasting responses to chemotherapy.

## Introduction

Many people in the world are battling cancer (Dolgin, 2021). The explicit pathogenesis of cancer has always been a hot topic in cancer research. Multiform therapies like chemotherapy, radiotherapy, targeted therapy, and immunotherapy are separately or compositely used clinically. Chemotherapy is a basic approach to treating cancer. Immunotherapy has greatly revolutionized both the research and treatment of cancer (Szeto and Finley, 2019). However, due to the complexity of carcinogenesis, the efficacy of cancer therapies cannot be guaranteed. Unfortunately, they are not able to enhance the long-term prognosis of patients. Therefore, it is of great significance to analyze a potential biomarker gene at the pan-cancer level and explore its connections to the clinical outcome of cancer patients. Currently, the largest database of cancer genes is the Cancer Genome Atlas (TCGA) (https://www.cancer.gov/ccg/research/genome-sequencing/tcga). More than 11,000 tumors from 33 of the most prevalent forms of cancer have been analyzed, constituting a uniquely comprehensive, in-depth pan-cancer atlas that serves as an essential resource for the development of new therapies in the pursuit of precision medicine (Hoadley et al., 2018).

TUBB (β-tubulin) is a protein-coding gene, responsible for forming a heterodimer with α-tubulin and acting as a structural component of microtubules (Miller et al., 2010), which are a kind of long hollow polymers that are 25 nm wide and range in length from <1 µm to >100 µm (Goodson and Jonasson, 2018). Microtubules play a major role in controlling different aspects of cell architecture and function (Wu and Akhmanova, 2017), they are essential for motor-driven intracellular transport, interact with accessory proteins to assemble into larger structures, and coordinate with other types of cells as a mature network (Goodson and Jonasson, 2018). As a result, compounds that target microtubules can interfere with multiple vital cellular processes (Wordeman and Vicente, 2021), for example, they inhibit microtubule polymerization, destroy the dynamic imbalance of microtubules, damage spindles, block cell cycle, and cause tumor cell death. Such anti-tumor drugs are collectively called microtubule-targeting agents (MTAs), served as cancer therapy for many years, the first being paclitaxel, introduced in 1994 (Field et al., 2014). What’s more, recent studies report that alterations in the expression of certain tubulin isotypes and associated post-translational modifications (PTMs) have been observed in human cancers, however, the exact implications of whether and how the tubulin code can mediate the biological progress of cancer cells are not yet clear (Lopes and Maiato, 2020). Therefore, TUBB as a mediator, has been studied in the cancer research area. In osteosarcoma, TUBB has been identified as the significant survival-predicting factor (Shao et al., 2022). Alhammad R. found that, the overexpression of TUBB would lead to a worse prognosis in ERα-positive and better prognosis in ERα-negative breast cancer (Alhammad, 2022). TUBB plays an important role in cancer progression and targeting TUBB may provide significant clues in cancer treatments. However, how TUBB modulates cancer initiation and advancement in pan-cancers remains controversial. Hence, it is necessary to explore TUBB profiles from the perspectives of multiple cancer cells. Thorough analyses are needed to understand the intrinsic role of TUBB in tumor immunity.

Considering the lack of pan-cancer analysis of TUBB, this study aims to conduct a comprehensive exploration of the potential roles of TUBB in malignant tumor cells and its underlying mechanisms in the prediction of clinical prognosis. The TUBB profiles, including their expressions, mutations, relations with aggressive tumor traits, and contributions to the survival of cancer patients, have been depicted. With the aid of several famous public databases, the analysis was performed based on the web tools and “R” software. We found that TUBB was significantly correlated with various cancer characteristics, tumor immune microenvironment (TIME), drug resistance, and survival states. All the results highlighted the critical roles of TUBB in cancer and they contribute to further studies of TUBB-related molecular mechanisms and therapeutic development.

## Materials and methods

### Acquisition and organization of public data from different databases

**FIGURE 1:**
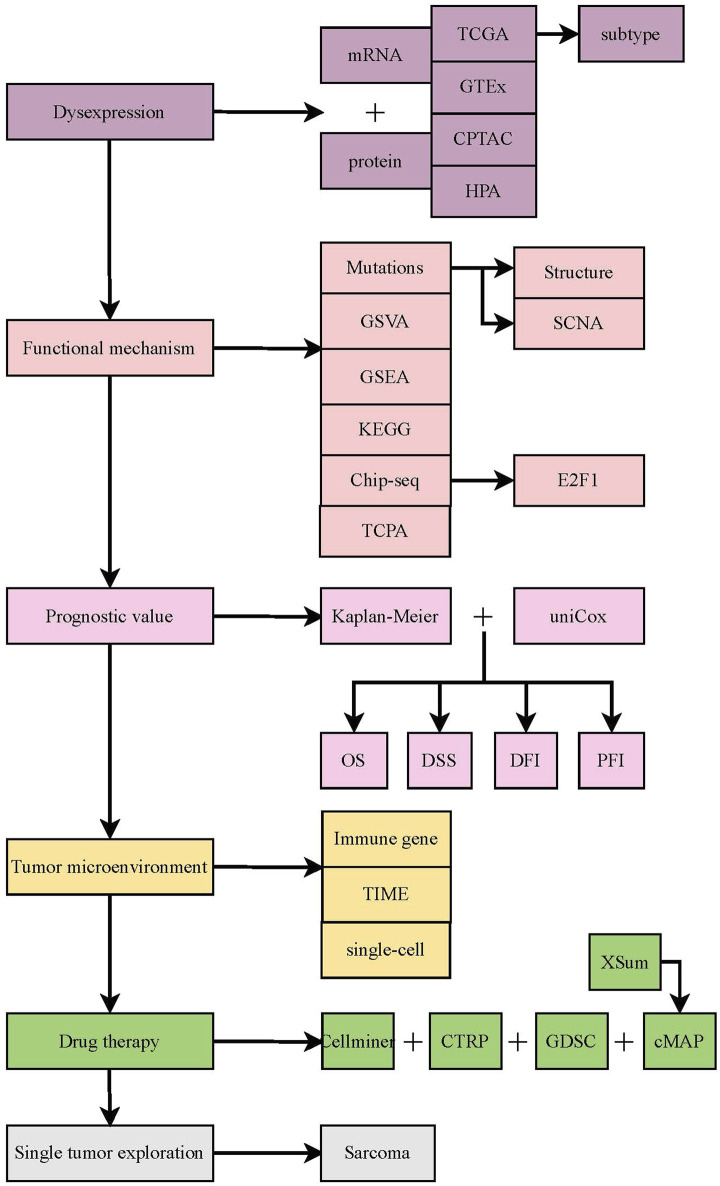
The flow chart of this study.

Firstly, the flow chart of this study is shown in Figure 1. Then, the mRNA expression data, copy number alteration threshold data, masked copy number segmentation data, and DNA methylation 450K data of both tumor and normal tissues in the TCGA pan-cancer cohort were obtained from the Firehose database (http://gdac.broadinstitute.org) (Deng et al., 2017). TCPA, mutation, and clinical data were acquired from the UCSC Xena database (https://xenabrowser.net/datapages/) (Wang et al., 2022). The mutation frequency of TUBB in the TCGA cohort was calculated using the cBioPortal database (https://www.cbioportal.org/) (Cerami et al., 2012). Based on the data from the GEO database (https://www.ncbi.nlm.nih.gov/geo/) (Barrett et al., 2013), the TUBB expression at the transcript level was validated. We analyzed and estimated its expression at the protein level based on the CPTAC database (https://proteomics.cancer.gov/programs/cptac) (Zhang et al., 2014). Also, various immune infiltrating algorithms from the TIMER2.0 database (http://timer.cistrome.org) (Li et al., 2020) were adopted to depict the correlations between TUBB expression and tumor immune microenvironment (TIME). Via 99 single-cell datasets from the TISCH database (http://tisch.comp-genomics.org/home) (Sun et al., 2021), immune infiltration results by whole transcriptome analysis were verified. From the CellMiner (https://discover.nci.nih.gov/cellminer/CellMiner) (Shankavaram et al., 2009), GDSC (https://www.cancerrxgene.org/) (Yang et al., 2013), and CTRP (http://portals.broadinstitute.org/ctrp/) databases (Rees et al., 2016), we gained relevant chemotherapy data to illustrate how TUBB expressions interact with drug sensitivity. It should be noted that these public databases are free and open. The study strictly follows the data extraction policy of the databases and does not require the ethical review and approval of the ethics committee.

### Differential expression analyses at multi-omics level

To observe whether imbalanced expressions of TUBB exist between tumor and normal tissues, differential analyses were conducted in terms of three dimensions.

First of all, we combined the data from the Genotype-Tissue Expression (GTEx) project because the TCGA-data was inadequate, to enhance the confidence by expanding the sample sizes. The “wilcox” test was applied to detect the variability (*p* < 0.05 was considered significant). The “gganatogram” R package was adopted to visualize the expressions in human’s different organs. Then, the “wilcox” analysis was performed to compare the differential mRNA expressions of TUBB between tumor and normal tissues based on TCGA data. Moreover, we performed the “wilcox” test on paired samples from the TCGA cancer groups (*p* < 0.05 was considered significant, *p < 0.05; **p < 0.01; ***p < 0.001; ****p < 0.0001). Using the “pROC” R package, the importance of TUBB in the diagnosis of pan-cancers was addressed. The area under curve (AUC) values were calculated (between 0.5 and 1, the closer the AUC value came to one, the better diagnostic performance TUBB would have). Usually, AUC values of 0.5–0.7, 0.7–0.9, and >0.9 indicate low accuracy, certainty accuracy, and high accuracy, respectively.

Based on the GEO database, we conducted the external validation at the transcriptional level. At the protein level, the protein expressions of TUBB were validated via the CPTAC database. What’s more, the immunohistochemical (IHC) staining of tissues was collected from the HPA database. The “Kruskal” test was used to identify the expressions of TUBB in different stages or molecular subtypes.

### Analyzing the survival status and clinical outcomes in pan-cancer

Survival data was obtained from the TCGA database, the “survival”and “survminer” R packages were utilized to describe the relations between the expression of TUBB and prognostic indexes of TUBB (including overall survival (OS), disease-specific survival (DSS), progression-free interval (PFI), and disease free interval (DFI)). Integrating the “Kaplan-Meier (KM)” analysis and the univariate COX analysis, we evaluated whether the TUBB was a protective or risky factor, and finally, the survival landscape of TUBB was drawn with a high level of confidence. Noticeably, the optimal cut-off between the high- and low-TUBB mRNA expression groups was determined via the “survminer” R package when the KM analysis was conducted. The “survfit” function was applied to conduct the “log rank” test to estimate the significance of the high- and low-TUBB expression groups. At last, the “forestplot” R package was used to visualize the results of survival data by the COX analysis.

### Somatic copy number alteration (SCNA), mutation, and DNA methylation analysis

The cBioPortal (http://www.cbioportal.org) website is a powerful tool for retrieving, downloading, analyzing, and visualizing cancer genomic data from various types of genomic data such as somatic mutations, DNA copy-number alterations (CNAs), and DNA methylation. It can undertake multiple analyses, including mutation analysis and its visualization (Cerami et al., 2012). On cBioPortal, “TCGA Pan Cancer Atlas Study” was selected in the “Quick Search” section (Gao et al., 2013), and “TUBB” was typed to search for its genetic altering traits. The mutation frequency, types, and CNA data were collected from the “Cancer Type Summary” part. The mutation locations of TUBB were shown in a two-dimensional (2D) diagram of the protein structure using the “mutation” function. Somatic copy number alterations (SCNAs) and mutations can increase the CNA of the gene by amplifying and deleting the heterozygosity and homozygosity. Generally, high-frequency SCNA is defined with >5% mutation frequency. Next, the “Spearman” correlations between the expression level of TUBB and CNA scores were calculated to estimate the relation between SCNA and TUBB expression. The “IlluminaHumanMmethylation-450kanno.ilmn12.hg19” R package from “Bioconductor” was conducted to annotate the methylation probe of the TUBB promoter. Through the “Wilcoxon” rank test was performed to detect the differential methylation of TUBB between tumor and normal tissues. A *p*-value cutoff of 0.05 was used to identify cancers that were significantly hypomethylated or hypermethylated. The Spearman correlation between TUBB expressions and promoter DNA methylation Beta was calculated (*p*-value < 0.05 was considered significant).

### Exploring pathways and functional mechanisms

To understand TUBB-related pathways, we divided tumor samples of each type according to TUBB expressions (top 30% and bottom 30%). Then the Gene Set Enrichment Analysis (GSEA) was carried out to compare differential activation or inhibition conditions of 50 hallmark gene sets and 83 metabolic gene sets in different tumors between the high- and low-TUBB group. Yuan H et al. have redefined 14 functional states of malignant tumoral features (Yuan et al., 2019). And, the “z-score” algorithm was proposed by Lee et al. (2008). It can reflect the activity of a given pathway by integrating expressions of characteristic genes. Using the “GSVA” R package, we conducted the “z-score” algorithm on the 14 functional state gene sets. The values were set as the z-score of each gene set. Then, the “Pearson” correlation analysis was performed to calculate statistical relations between TUBB expression and the z-score of each gene set. Additionally, we identified the differential genes between the high- and low-TUBB group. Moreover, to search for transcription factors that may affect the expression of TUBB, we used the CistromeDB database (http://dbtoolkit.cistrome.org/) to identify potential regulatory upstream factors for TUBB. As known, protein-protein interacting data usually contains those unlikely biological interactions that are impossible to happen in living cells. Therefore, we used the ComPPI database (https://comppi.linkgroup.hu/) which introduces two novel quantitative scores, the Localization Score and the Interaction Score, describing the calculated probability of the data correctness, to gain the proteins that interact with TUBB. TUBB has been identified as a risky factor according to four survival indexes in sarcoma (SARC). Hence, we conducted the GSEA on multiple gene sets to analyze possible pathways that SARC may participate in. It was believed that if one gene had differential expression in more than five cancers, its functional gene would be thought to be related to TUBB and undertake a KEGG enrichment analysis to recognize the conservative functions or pathways that TUBB takes part in pan-cancers. Subsequently, we adopted the permutation test to identify the mutation that was statistically related to TUBB expressions in SARC. Then, using Fischer’s exact test, we estimated the relationship between E2F1 and TUBB expressions. In the end, we conducted the Spearman correlation analysis to systemically identify BEND3-related proteins.

### Exploring the tumor microenvironment and performing a single-cell analysis

The continuous interactions between tumor cells and the tumor microenvironment (TME) play a decisive role in tumor initiation, progression, metastasis, and responses to therapies. As a consequence, we confirmed correlations between TUBB expressions and immune-related genes (immunostimulatory genes, immunoinhibitory genes, chemokine genes, chemokine receptor genes, and MHC genes). We also adopted the same approach to conduct the differential analysis on TIP scores. Next, from the TIMER2.0 databases, seven most advanced algorithms were applied (CIBERSORT, CIBERSORT_ABS EPIC, MCPCOUNTER, QUANTISEQ, TIMER, XCELL) to estimate the immune filtration profiles of TCGA cancers (Li et al., 2020). At last, we used the TISCH database to download the expression landscape of TUBB in 99 single-cell datasets of 38 tumors, which helped us verify the TME analytic result at the single-cell level. In summary, we provided a comprehensive analysis and visualized the TME and immune infiltration profiles of TUBB in a pan-cancer cohort. It is worth noting that, we analyzed the anti-tumor immunity in seven steps of the cancer-immunity cycle (release of cancer cell antigens, cancer antigen presentation, priming and activation, trafficking of T cells to tumors, infiltration of T cells into tumors, recognition of cancer cells by T cells, killing of cancer cells (Chen and Mellman, 2013) and conducted the Gene Set Variation Analysis (GSVA) on each step, and then we compared their differences in the high- and low-TUBB groups.

### Identification of chemical substances interacting with TUBB

Based on the GSCA database (http://bioinfo.life.hust.edu.cn/web/GSCALite/), we analyzed the relations between TUBB expressions and drug sensitivity (Liu et al., 2023). GSCA website contains 750 small molecule drugs from GDSC and CTRP databases. Besides, the gene expression data is used to mine the valuable small molecule drugs related to it. In addition, the National Cancer Institute (NCI) database was utilized. NCI serves as a trusted source of cancer information, and the platform of cancer cell lines from NCI has been widely used to screen out drugs that are related to a certain gene’s expression. NCI-60 is a collection of 60 cancer cell lines from nine different kinds of cancers (leukemia, colon, lung, central nervous system, kidney, melanoma, ovarian, breast, and prostate cancers). Its data comes from the “CellMiner” database (Reinhold et al., 2023), for us to analyze the Spearman correlations between TUBB mRNA expressions and the z-score of drug sensitivity. Moreover, differentially expressed genes between the high- and low-TUBB group in different cancers were identified. We collected the top 150 upregulated and downregulated genes as TUBB-related biomarkers. The CMAP_gene_signatures.RData document containing 1,288 compound-related characteristics was downloaded from the BHKLAB database (https://www.pmgenomics.ca/bhklab/sites/default/files/downloads) and used to calculate matching scores. All analyzing processes kept to the methods from the previous literature (Malta et al., 2018). The R software was used to summarize and display the top five results of 32 kinds of cancers.

### Statistical analysis

All data was processed using the web tools and R software (V.4.3.0, Institute of Statistics and Mathematics, Vienna, Austria). The Pearson correlation analysis was used on normally distributed data, otherwise the Spearman correlation analysis was used. The Kruskal–Wallis rank sum test, Wilcoxon rank sum and Signed rank tests were used to detect the differences between multiple variables or two variables, respectively. Using the “survival” R package, the COX and KM survival analyses were conducted. KM method adopted the log-rank test to detect significance. The “survminer” R package was used to visualize results from KM analysis. Hazard ratio (HR) and 95% confidence interval (CI) were used to describe relative risks. The “pROC” R package was utilized to perform ROC analysis to estimate the diagnostic ability of TUBB. All statistical tests were two-tailed. *p*-value < 0.05 was deemed as statistically significant. *p*-value < 0.0001 was deemed as greatly statistically significant (*p < 0.05, **p < 0.01, ***p < 0.001, and ****p < 0.0001).

## Results

### Aberrant expressions of TUBB among cancers

**FIGURE 2:**
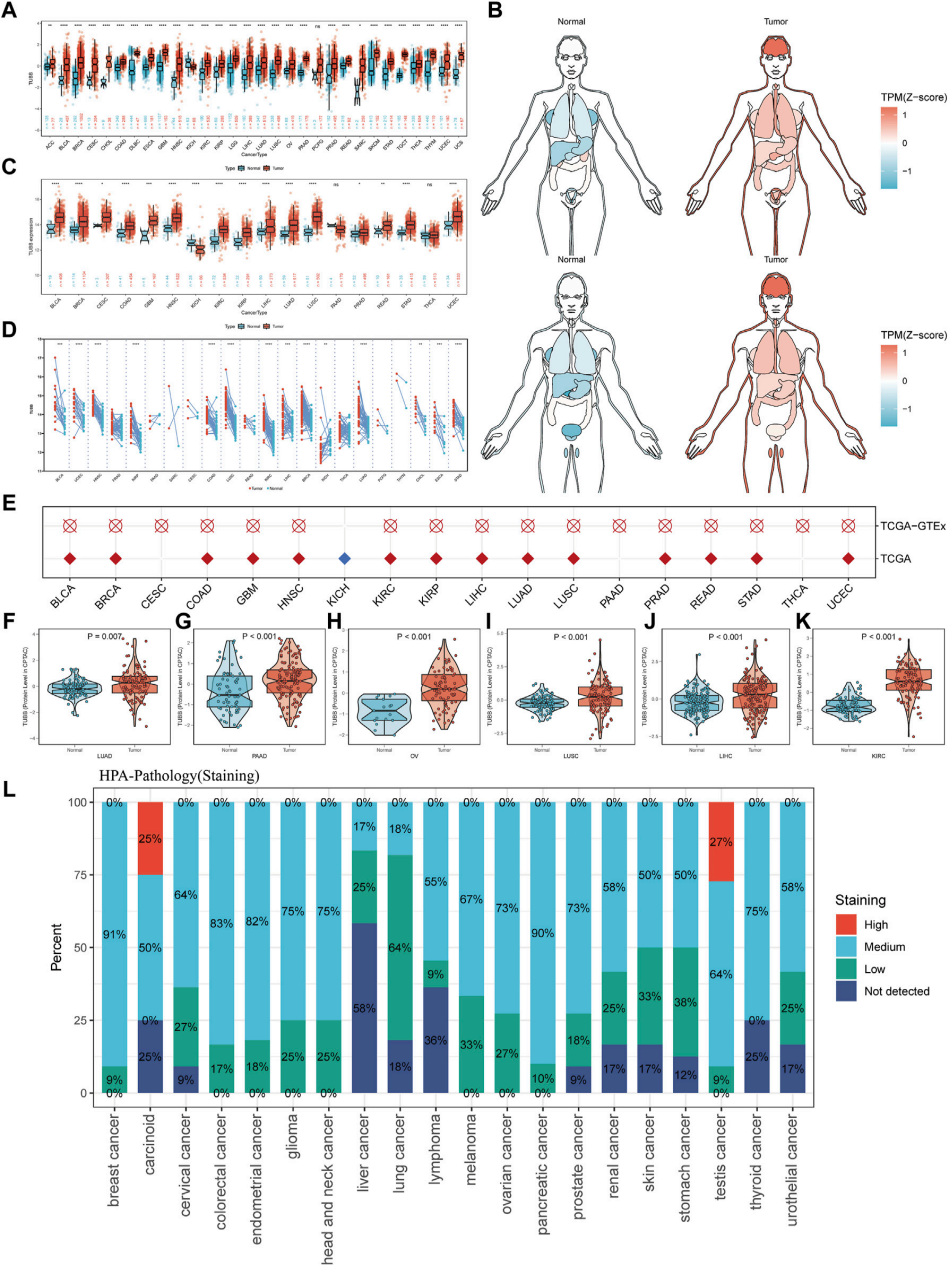
The expression profiles of TUBB in pan-cancer. **(A)** TUBB expressions in between tumor and normal tissues in various cancers using TCGA and GTEx data. **(B)** Expressions and distributions of TUBB between tumor and normal tissues in various organs. **(C)** TUBB mRNA expressions in the TCGA between tumor and normal tissues in TCGA. **(D)** Similar to **(C)**, but in paired samples grouped by cancer from TCGA. Each point representing one sample. (*p < 0.05, **p < 0.01, ***p < 0.001, ****p < 0.0001) **(E)** Logistic regression analysis of TCGA, TCGA-GTEx. Red means OR>1, blue represents an OR value between 0 and 1. White circle means no significance. **(F–K)** Differential protein levels between tumor and normal tissues in LUAD, PAAD, OV, LUSC, LIHC, and KIRC based on the CPATC database. **(L)** IHC results indicate that TUBB showed weak to moderate cytoplasmic positivity in most malignant cells. Different colors represent different staining indicators.

**FIGURE 3:**
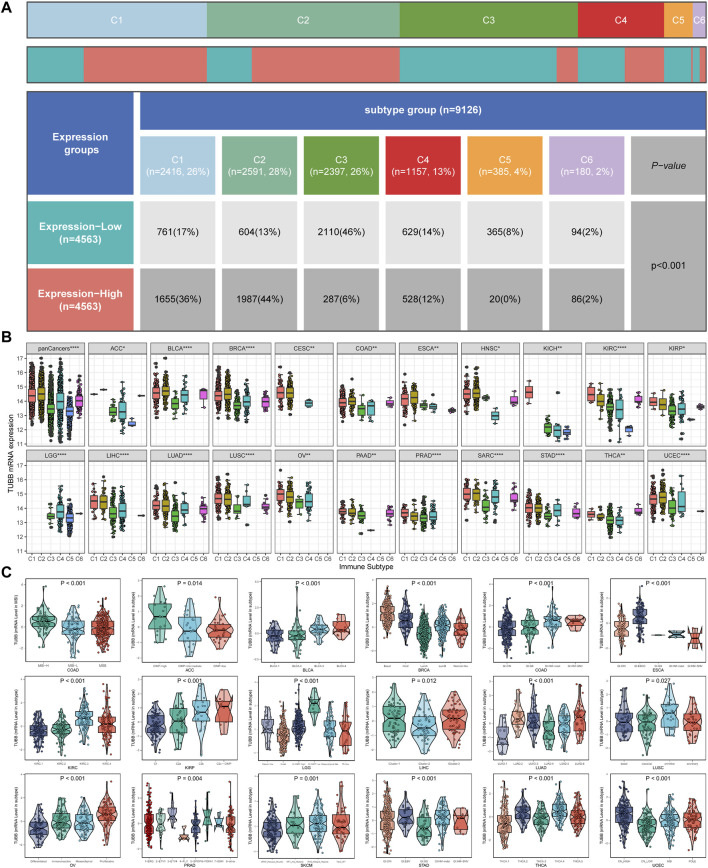
Analysis of clinical variables and molecular subtypes. **(A)** More patients with C1 and C2 immune subtypes in the TUBB high-expression group and more patients with C3 subtypes in the TUBB low-expression group. **(B)** Expression levels of TUBB in different immune subtypes. C1 (wound healing); C2 (IFN-gamma dominant); C3 (inflammatory); C4 (lymphocyte depleted); C5 (immunologically quiet); C6 (TGF-b dominant). The Kruskal test detects differences between six immune subtypes. **(C)** Differences in TUBB expression in different molecular subtypes.

To identify patterns of TUBB regulations in cancers, we combined TCGA and GTEx data to expand sample sizes and gained a boxplot describing TUBB expressions in pan-cancer (Figure 2A). Figure 2B showed TUBB expressions in different organs. TUBB had a differential expression in most cancers. It was greatly upregulated across cancers. Then, the differential analysis based on TCGA samples and paired samples were shown in Figures 2C, D. With the aid of logistics regression analysis using TCGA and TCGA-GTEx data, above results were validated (Figure 2E). An external validation of TUBB mRNA expressions was based on GEO database (Supplementary Figure S1). From CPTAC database, the validation at the protein level was conducted (Figures 2F–K). It was observed that results from different angles and databases had good consistency. IHC results showed that, most cancer cells displayed weak to moderate cytoplasmic immunoreactivity. A few seminomas and carcinoids were strongly stained. Most hepatocellular carcinomas were negative (Figure 2L). Moreover, TUBB expressions were correlated with tumor stages in seven cancers (ACC, ESCA, KIRC, KIRP, LIHC, SKCM, STAD) (Supplementary Figure S2), implying that TUBB may have a relationship with the progression of some cancers. The estimated ROC curves showed that (Supplementary Figure S3), the TUBB mRNA expression level showed satisfactory sensitivity and specificity for the diagnosis of nine kinds of tumors (AUC>0.7). After expanding normal sample sizes with GTEx data, the results also were robust (Supplementary Figure S4). The repeatable and consistent results were demonstrated across multiple databases, multiple tumors, multiple methods, and multiple omics, suggesting that dysregulation of TUBB expression may play a crucial role in different cancers and was highly unlikely to be a false finding due to technical artifacts, chance, or bias in the eligibility criteria for TCGA samples. In addition, by the chi-square test, we found that C1 and C2 immune subtypes were more in the high-TUBB group (Figure 3A). The same results were obtained using the Kruskal test, that is, TUBB was expressed more in the C1 and C2 immune subtypes compared to other subtypes (Figure 3B). Interestingly, TUBB showed differences in a large number of molecular subtypes. For example, it had the lowest expression in LumA group, and in the basal group, its expression was the highest in breast cancer (BRCA), meaning that it had great values in the precision molecular stratification therapies and prognosis prediction (Figure 3C).

### Clinical relevance of TUBB

**FIGURE 4:**
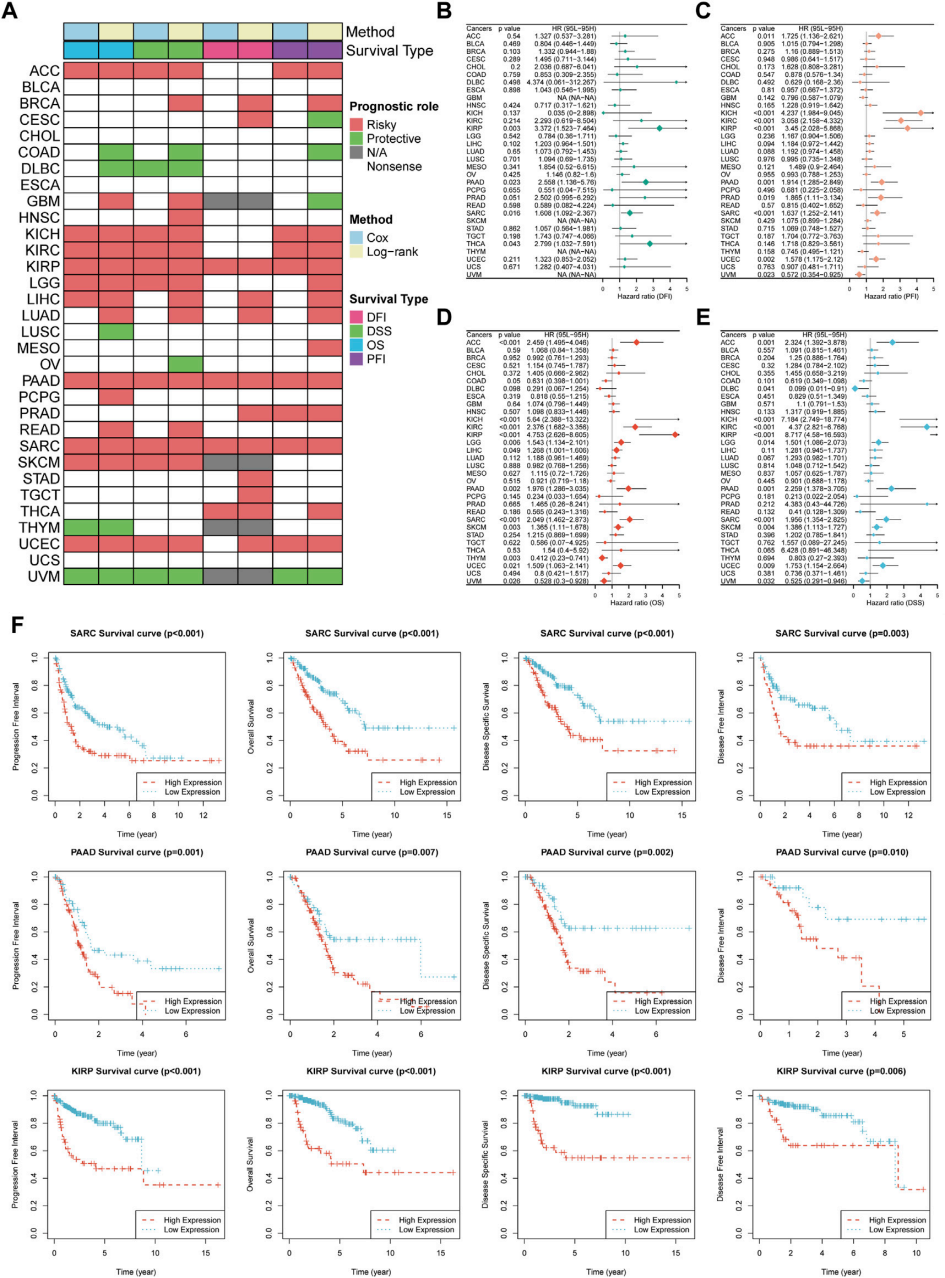
Survival profiles of TUBB in pan-cancers. **(A)** Correlations between TUBB expressions with overall survival (OS), disease-specific survival (DSS), disease free interval (DFI) and progression-free interval (PFI) based on the univariate Cox regression and Kaplan-Meier models. Red indicates that TUBB is a risk factor affecting the prognosis of cancer patients, and green represents a protective factor. Only *p* values <0.05 are shown. **(B–E)** Forest plots exhibit the prognostic role of TUBB in cancers by univariate Cox regression method. **(F)** Kaplan-Meier survival analysis and log-rank test were performed using “survival” and “survminer” packages.

To further dissect the clinical relevance of TUBB in cancers, the role of TUBB was analyzed. Survival profiles of pan-cancers showed that TUBB expressions were related to many kinds of survival indicators in pan-cancers (Figure 4A), and the relations had homogeneity. TUBB was identified as a risky factor in cancers, especially in KIRP, PAAD, and SARC because TUBB was risky weighed by all indicators DFI, DSS, OS, and PFI. In several cancers, TUBB was protective. For example, UVM patients with higher TUBB expressions would have better survival probability. Due to the highly heterogeneity across various cancers, in some cancers (BLCA, CHOL, ESCA, UCS), the tendency was vacant. Therefore, TUBB may play different roles in pan-cancers, suggesting further explorations of it should be addressed. Forest plots showed COX survival analysis results of four survival indicators. The hazard ratios of each cancer were listed, too (Figures 4B–E). KM curves were used to display the log-rank test results in KIRP, PAAD, and SARC (Figure 4F).

### Genetic alterations of TUBB in cancers

**FIGURE 5:**
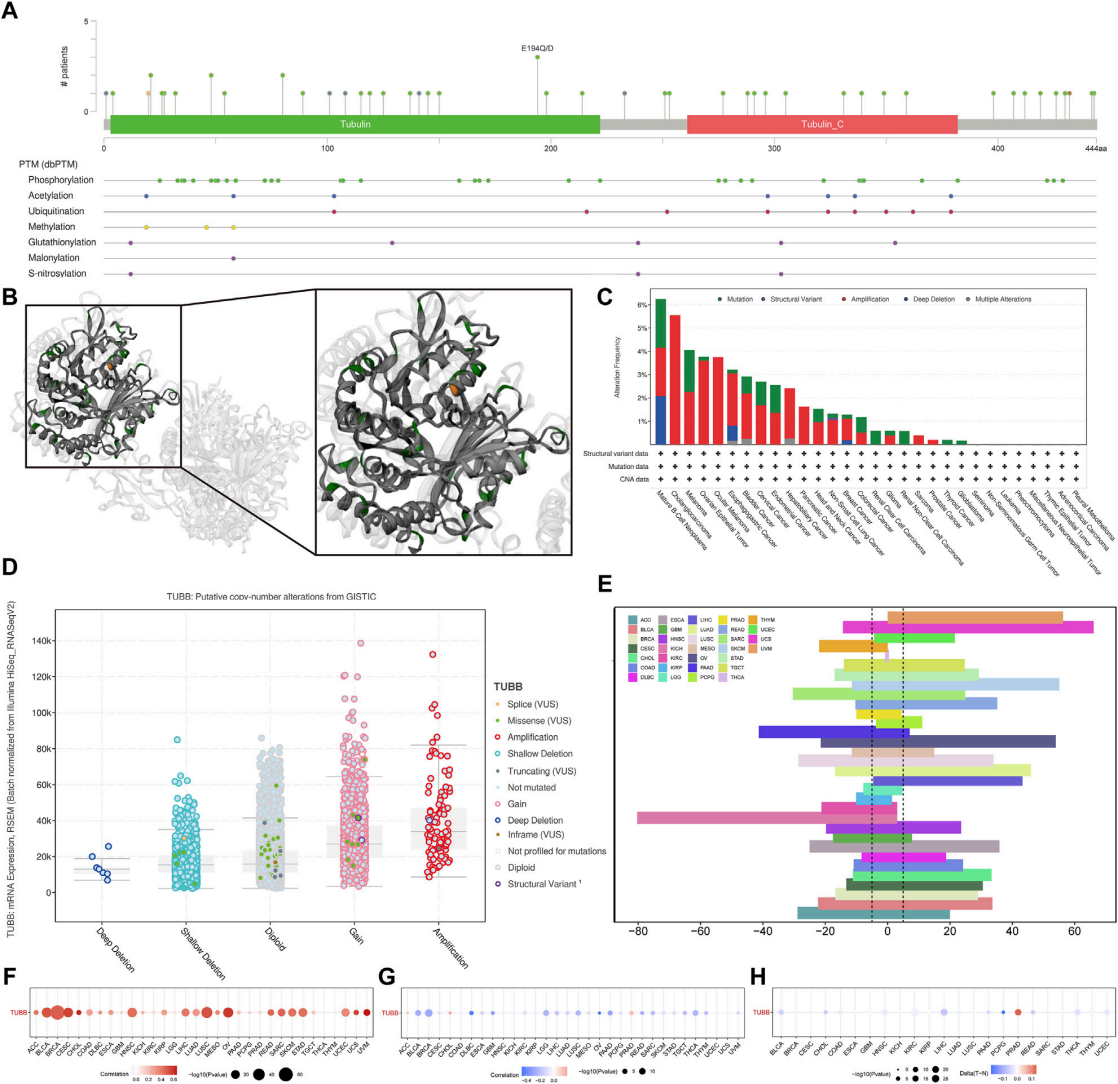
Genetic alterations of TUBB in cancers. **(A)** Sites and numbers of TUBB genetic alterations across cancers from cBioPortal. **(B)** 3D structure of TUBB mutation sites. **(C)** Frequency of TUBB mutations in different tumor types. **(D)** Relationship between TUBB mRNA expressions and genetic alterations. **(E)** Histogram shows the frequency of somatic copy number alterations for TUBB in each cancer type. **(F)** The Spearman’s correlation between somatic copy number alterations and the expression of TUBB. **(G)** Spearman’s correlation of TUBB between transcriptional expressions and promoter methylation. Red and blue represent positive and negative correlations, respectively. **(H)** Bubble Gram shows the differential methylation of TUBB in cancers; hypermethylated and hypomethylated TUBBs are marked in red and blue, respectively (Wilcoxon rank-sum test).

We analyzed genomic data (genetic variation, somatic copy number alteration (SCNA), mRNA expression, and DNA methylation) of tumor and normal tissues from the TCGA cohort. Mutation sites of TUBB were visualized in 2D and 3D graphics (Figures 5A, B). TUBB had genetic changes in most cancers, and the most common alteration types were amplification and mutation (Figure 5C). Obviously, SCNA plays an essential role in regulating gene expression in cancers (Figure 5D). To study genetic changes of TUBB in cancers, we checked the percentage of SCNA. SCNA appeared frequently in most cancers (more than 5% among all samples). Only in a few types, the frequency was low (Figure 5E). Therefore, we estimated how SCNA affected TUBB mRNA expressions by calculating the Spearman correlation between TUBB expressions and masked copy number segment of TCGA. In most cancers, the mRNA expression of TUBB was significantly correlated with SNCA (Figure 5F). Therefore, it could be concluded that TUBB CNAs were common among cancers, and it could affect TUBB expressions. Besides, promoter methylation of TUBB can also regulate gene expressions. And, abnormal DNA methylation of promoters was associated with tumorigenesis. The methylation patterns of TUBB in pan-cancers were consistent with each other. From all results, it was observed that TUBB mRNA expressions were negatively correlated with DNA methylation (Figure 5G), and higher methylations existed in tumor tissues more than in normal tissues in most cancers (Figure 5H) because when TUBB is significantly overexpressed, the body increases promoter methylation to overcome this dysregulation to keep the balance. Great differences in the methylation patterns of TUBB suggested the complexity of TUBB regulation and the specificity of this process among different cancer species. In addition, we selected 10 transcription factors with the highest scores to annotate peaks and found that they were usually located in the promoter region of TUBB, showing that they may regulate the expression of TUBB.

### Associations between TUBB and cancer-related pathways

**FIGURE 6:**
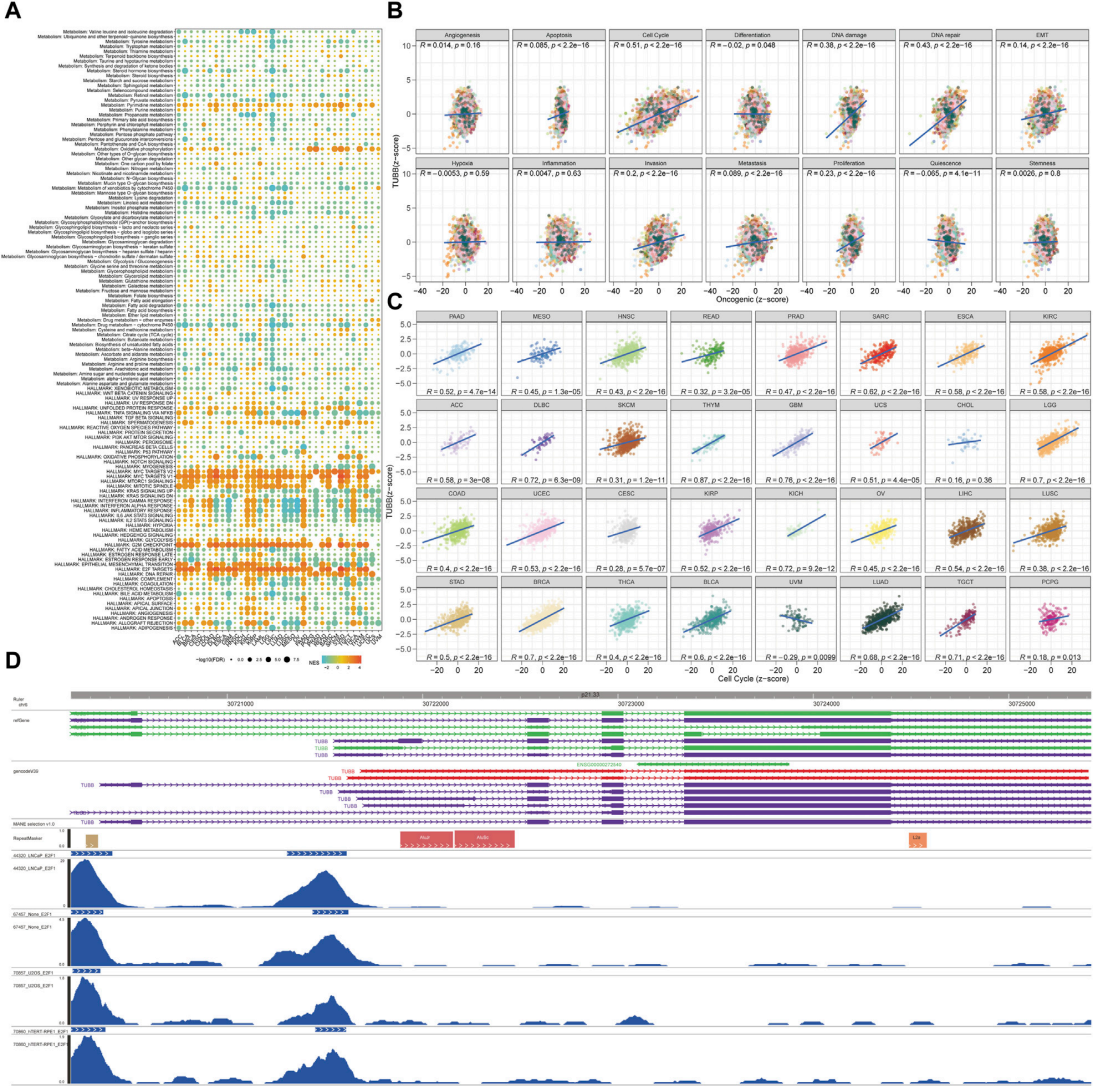
Analyses of pathways and underlying mechanisms. **(A)** Enrichment differences of TUBB in 50 HALLMARK and 83 metabolism gene sets. **(B)** The TUBB mRNA expression was highly correlated with 14 malignant features of all tumors. **(C)** The TUBB mRNA expression was highly correlated with cell cycle features of all tumors. **(D)** The 10 transcription factors with the highest scores were selected for peaks annotation, and it was found that they were usually located in the promoter region of TUBB.

**FIGURE 7:**
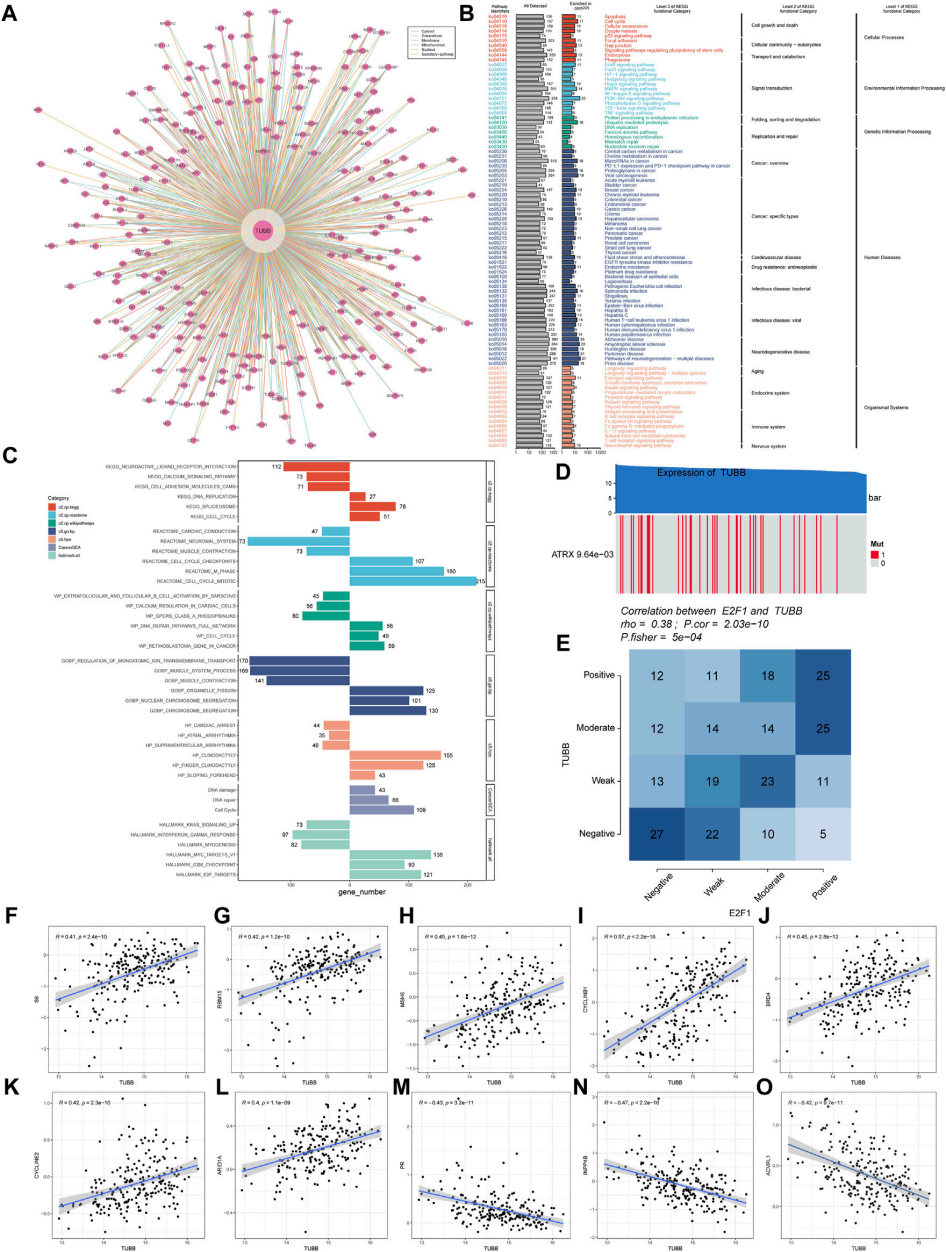
Deeper exploration of TUBB mainly in SARC. **(A)** Networks of genes interacting with TUBB. **(B)** KEGG enrichment analysis results showing highly expressed genes. **(C)** GSEA enrichment analysis results. Different colors represent different gene sets. Bar charts on the left means significant enrichment in the high-TUBB group, and *vice versa*, significant enrichment in the low-TUBB group. **(D)** The value adjacent to highly mutated gene is permutatio test *p*-value of gene expression between driver mutated (red) and not-mutated (gray) samples. **(E)** Fischer’s precise test explores the correlation between E2F1 and TUBB. **(F–O)** TUBB related proteins of SARC in the TCPA database.

Samples with top 30% and bottom 30% TUBB expressions were set as the high- and low-TUBB group. Based on two groups, we conducted the GSEA to explore relevant cell signaling in cancers. Furthermore, metabolism-related pathways were analyzed systematically, and the results had satisfying consistency across cancers, indicating the functions of TUBB were highly conservative. What’s more, pathways related to cell-cycle were enriched in tumors with higher TUBB. Besides, TUBB may take part in metabolic disorder processes in cancers. Meanwhile, TUBB was believed to inhibit the course of drug metabolism, thus it may be related to responses to chemotherapy (Figure 6A). We analyzed the correlations between z-scores of 14 symbolic functional states of cancers (angiogenesis, apoptosis, cell cycle, differentiation, DNA damage, DNA repair, EMT, hypoxia, inflammation, invasion, metastasis, proliferation, quiescence, stemness) with the z-score of TUBB expressions. Among them, the z-score of the cell cycle had the highest R-value (0.51), showing a positive relation with TUBB expressions (Figure 6B). Then, in multiple cancers, we ran a Pearson correlation analysis between the z-score of TUBB expressions and the z-score of the cell cycle state. Learning from Figure 5C, in thymoma (THYM) the highest coefficient of 0.87 was detected (Figure 6C). CHIP-seq demonstrated that E2F1 might be an upstream transcription factor modulating the TUBB expression (Figure 6D). ComPPI helped identify the genes that may interact with TUBB (Figure 7A). The KEGG enrichment analysis was conducted on genes that were highly expressed in the high-TUBB group. Figure 7B showed that TUBB had relations with a lot of functions, especially with signal transduction, immune, and cancer-related pathways. As previously mentioned, TUBB was determined as a risky factor in terms of four survival indexes in SARC. Then we performed the GSEA on various gene sets to fully analyze the pathways that TUBB may be involved in (Figure 7C). From Figure 7D, the expression of TUBB probably was relevant to ATRX mutations. In SARC, E2F1 was statistically associated with TUBB expressions (Figure 7E). Besides, based on the TCPA database, some TUBB-related proteins were identified (Figures 7F–O). Among them, CYCLINB1 had the most significant relation with TUBB mRNA expressions with the highest coefficient of 0.57.

### High TUBB expression correlates with immune infiltration in cancers

**FIGURE 8:**
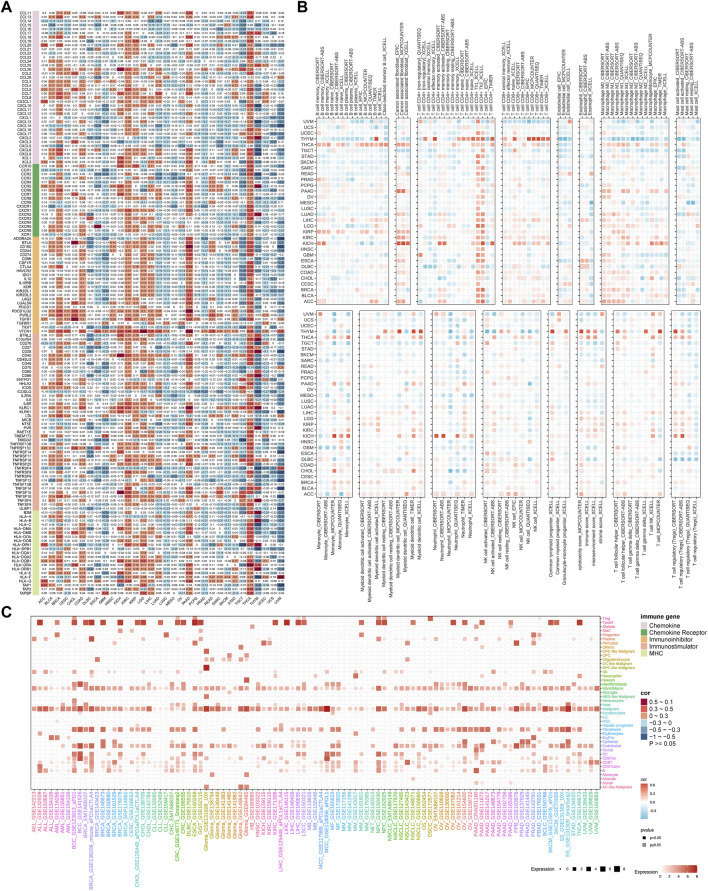
Association of TUBB expressions with immune infiltration. **(A)**. The heatmap shows correlations between TUBB mRNA expressions and expressions of chemokine, chemokine receptor, immune-inhibitor, immune-stimulatory, and MHC genes. **(B)** Seven software were used to evaluate the correlations between TUBB expression and cancer immune infiltration. **(C)** Cell sources of TUBB in pan-cancer at the single-cell level.

First of all, the heatmap displayed the landscape of immune infiltration of TUBB across cancers (Figure 8A). TUBB was significantly negatively correlated with immune-related genes (MHC, immune-inhibitor, immune-stimulator, chemokines). Notably, we scored seven steps and compared the differences between the high- and low-TUBB group, and the tendency was consistent in pan-cancers, namely the TIP scores were lower (Supplementary Figure S5). To illustrate cells regulated by TUBB in the TME, we used the TIMER2.0 database to explore correlations between TUBB mRNA expressions and immune infiltration and stroma cell abundance (Figure 8B). For example, in almost every cancer the TUBB mRNA expression level was positively related to the abundance of CD4^+^ Th1 cells. In SARC, the TUBB mRNA expression level was negatively related to the abundance of B cells and CD8^+^ T cells across different software, which could possibly explain why TUBB was risky in cancers. Different proportions of immune infiltration and unique TMEs in pan-cancers existed, so the correlations showed various change laws. However, we utilized seven different algorithms to conduct the analysis and made the results verify each other to ensure the accuracy of the study. Additionally, we could see from Figure 8C that the single-cell analysis showed that though TUBB was not expressed strikingly in most tumors, it mainly originated from malignant cells and proliferative T cells. This echoed previous results. In other words, TUBB might encourage the formation of immunological rejection or “immunological desert”, and play a vital role in immunity-cancer crosstalk, especially in immune escape.

### TUBB may influence responses to chemotherapy

**FIGURE 9:**
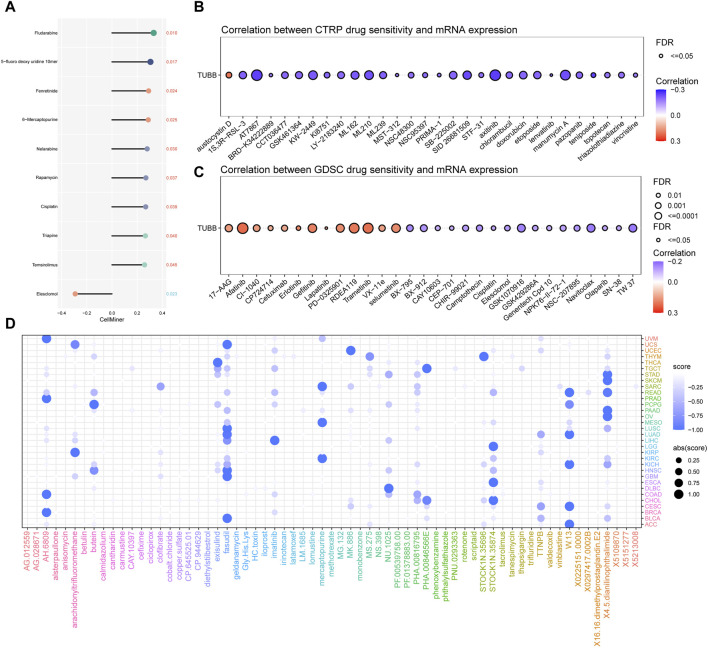
Chemotherapeutic drug resistance analysis. Correlations between TUBB expressions and drug sensitivity using the three different databases Cellminer **(A)**, CTRP **(B)**, GDSC **(C)** (*p* < 0.05 was considered statistically significant). **(D)** Prediction of potential compounds targeting TUBB.

Using Cellminer data, we found that TUBB was positively related to the sensitivity of a lot of drugs (Figure 9A), but negatively related to the half maximal inhibitory concentration (IC50) values of many drugs based on CTRP, GDSC databases (Figures 9B, C). Therefore, we could deduce that TUBB was a potential chemotherapy-sensitive gene. To further explore underlying therapeutic regimens that offset the tumorigenic effect mediating by TUBB, CMap analysis was employed. Thus, a TUBB-related signature containing 150 significantly upregulated and 150 significantly downregulated genes that were selected by screening patients with high- or low-TUBB expressions in various cancers, was generated. Using the Extreme Summarization (XSum) method, TUBB-related traits were compared with CMap gene traits, and the similarity scores for 1,288 compounds were obtained. Fasudil, W.13, AH.6809 and X4.5. dianilinophthalimide showed relatively lower scores in most cancers, which demonstrated that they perhaps inhibit TUBB-mediated carcinogenic effects (Figure 9D). These findings provided substantial support for the validity of our predictions, although further researches are needed to elucidate the underlying mechanisms.

### TUBB expressions in the tissue sample

**FIGURE 10:**
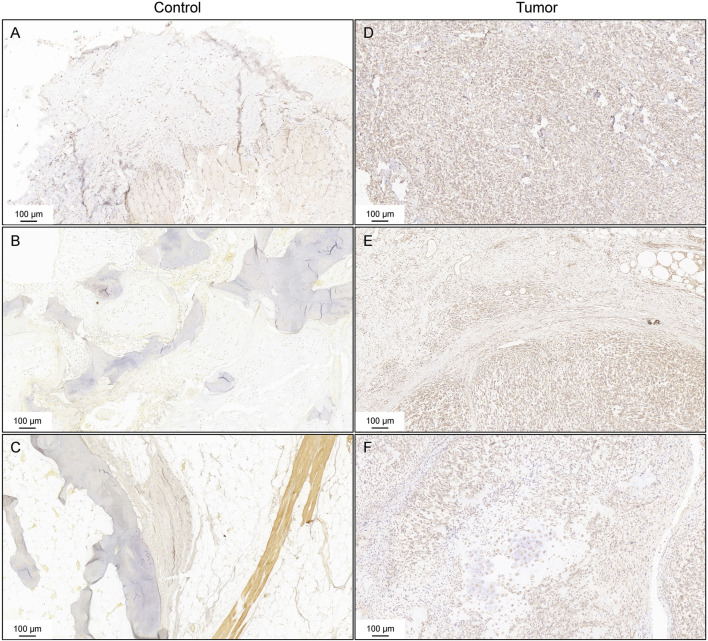
Immunohistochemistry staining for TUBB. Negative controls of the expression of TUBB **(A–C)**. IHC staining of osteosarcoma tissues **(D,E)**. IHC staining of chondrogenic sarcoma tissues **(F)**.

To verify the expression of TUBB among the tissue samples of cancer patients, IHC staining was conducted. Figure 10A–C displayed the negative results of TUBB expression in the control subjects. TUBB exhibited strong diffuse staining in osteosarcoma tissues (Figures 10D, E). Positive staining was also found in chondrogenic sarcoma tissues (Figure 10F).

## Discussion

Hitherto, cancer-related research has always been a main point and difficulty in the current domain. With data from multi-platforms, multi-omics approaches were used in this study. TUBB was identified as an important indicator across multiple cancers. TUBB was valuable in the early-detection, prognosis prediction, therapy selection in pan-cancers. Also, we found that TUBB was related to many vital biological pathways, indicating its indispensable roles in cancers. TUBB was validated as a risky factor in some cancers, and immune infiltration analyses showed it may perhaps induce immune escape in tumor cells. Genes related to TUBB were identified too. Besides, we explored how TUBB could potentially affect responses to chemotherapies, in order to provide practical clues for clinical use. Compounds that can target and reverse the tumorigenic functions of TUBB. All results generated by multiple biological methods mutually pointed to the research value of TUBB in the future. And more experiments are wanted to verify our findings.

TUBB (Tubulin β class I gene) refers to a class of genes that can encode tubulin, a fundamental structure of microtubule cytoskeleton (Janakiraman et al., 2023). Microtubules are major components that dynamically control many vital functions of cells ranging from cell division to cell movement and vesicular transport (Gudimchuk and McIntosh, 2021). Nowadays, robust therapeutic targets of cancer treatments are widely and profoundly sought, among all kinds, microtubule-targeting agents (MTAs) are emerging as a time-proven anti-tumor chemical (Čermák et al., 2020). MTAs can be divided into two main categories: microtubule-stabilizing agents (MSAs) (Zhao et al., 2016) and microtubule-destabilizing agents (MDAs) (Borys et al., 2020), and they have been studied in many cancers (Karahalil et al., 2019; Khwaja et al., 2021; Anwar et al., 2022; Chen, 2023), epithelial ovarian cancer (Tymon-Rosario et al., 2021), non-small cell lung cancer (NSCLC) (Tagliamento et al., 2019), *etc.* Therefore, as the microtubule-encoding gene, TUBB’s mediating functions in cancers should be noticed and clarified. MicroRNAs (miRNAs) are small endogenous non-coding RNAs that post-transcriptionally repress gene expression (Lu and Rothenberg, 2018). The miR-195 axis has been identified as a multipurpose bridge in regulating cancer processes including metastasis and chemoresistance. Yu et al. (2019) have demonstrated that TUBB is directly targeted by miR-195, and participates in regulating the response of lung adenocarcinoma cells to MTAs. What’s more, it has been experimentally verified that silencing LINC00665 contributes to melanoma cell viability decline, inhibited proliferation, migration, invasion, and cell cycle progression, and enhances apoptosis by regulating the miR-339-3p/TUBB axis (Liu Y. et al., 2022). However, the exact role of TUBB in pan-cancers has not been illustrated. Here we integrated multiple strategies and utilized pan-cancer data from public databases for further analysis. TUBB’s underlying functions across cancers have been uncovered to some extent, providing new insights for cancer profiling in depth.

First of all, we determined the expression levels of TUBB mRNA and protein in tumor tissues compared to that in normal tissues across various cancers. Results showed that TUBB was found to be generally highly expressed in tumor cells more than normal cells. At transcriptional and protein level, we proved TUBB was much closer to be a risky factor for cancer. ROC curves showed high confidence to again confirm the results above. We conducted mutation analyses to depict the mutation profiles of mutation types, frequency, and sites in pan-cancer. Generally speaking, copy number variations were commonly seen in cancer, and these could in turn affect TUBB expressions. And, we exhibited TUBB mutation sites in a vivid way with 2D and 3D diagrams. Furthermore, we explained the importance of TUBB using the concept “z-score”. We found that TUBB mRNA expressions were related to the z-score of 14 newly identified cancer-related function states. Among them, TUBB expressions was mostly positively related to the z-score of cell-cycle status, showing TUBB may participate in regulating the course of cell-cycle. Based on the z-scores of cell cycle, we analyzed their correlations with TUBB expressions in various cancers. Almost a positive tendency was observed in any cancer. In thymoma (THYM), the coefficient was the highest. Cell cycle is a highly controlled and regulated process enabling cell growth, duplication of genetic material, and cell division, aberrancy in its progression is one of the basic mechanisms underlying tumorigenesis, making regulators of the cell cycle machinery reasonable anticancer therapeutic targets (Suski et al., 2021; Liu J. et al., 2022; Matthews et al., 2022). Therefore, it was deduced that TUBB might be enrolled in modulating the process of cell cycle, and thus eventually have an effect on tumor cells. From immune infiltration analyses, we confirmed that TUBB plays an important role in regulating immunity across cancers. It was significantly negatively correlated with MHC, MHC, immune-inhibitor, immune-stimulator, and chemokines genes. Moreover, we utilized seven algorithms and found that in almost every cancer the TUBB mRNA expression level was positively related to the abundance of CD4^+^ T helper 1 (Th1) cells, characterized by the production of proinflammatory cytokine interferon-gamma (IFN-γ), plays a central role in orchestrating cell-mediated immunity against tumor cells (Yang et al., 2020). They are responsible for generating effector and memory cytotoxic T lymphocytes (CTL) in facilitating immune responses, so CD4^+^ Th1 cells are important targets in the field of tumor immunotherapy. In SARC, the TUBB mRNA expression level was negatively related to the abundance of B cells and CD8^+^ T which are both strongly immune-related cells and pivotal targets for cancer therapies (Wennhold et al., 2019; Chow et al., 2022; Lundberg et al., 2022). From the single-cell analysis, TUBB was demonstrated to mainly originate from malignant cells and proliferative T cells. All results mutually indicated that TUBB was wicked and may induce immune escape in cancer, causing indifferent immune responses to cancer treatments, resulting in poor clinical outcomes at last. Based on it, we explored chemotherapeutic drug resistance on a deeper level to try to understand the rule behind lower responses to medications. Drug sensitivity data from three relevant databases were gleaned. From various angles, we analyzed correlations between TUBB expressions and drug sensitivity. TUBB played a vital role in responses to drugs. However, the exact role of TUBB remained unclear. Barrón-Gallardo et al. (2022) used to elucidate that in chemotherapy-resistant breast cancer patients, higher TUBB expressions were observed. As a consequence, more studies should be emphasized to clarify TUBB functions in medication guidance. Next, we found that Fasudil, W.13, AH.6809, and X4.5.dianilinophthalimide might reverse TUBB-mediated carcinogenic effects. These results may inspire relevant researchers. In addition, deeper explorations of TUBB mainly in SARC were conducted. We identified E2F transcription factor 1 (E2F1) as having a statistical relation with TUBB expressions in SARC. E2F1 has been reckoned as a tumor-promoting gene. From one pan-cancer analysis, the KAT2A/E2F1 complex promotes cell proliferation and metastasis by upregulating the UBE2C expression (Lin et al., 2022). An in SARC, the role of E2F1 has already been widely explored. As previously reported, E2F1 will promote Warburg effect and cancer progression via upregulating ENO2 expression in Ewing SARC (Jiang et al., 2022). What’s more, MNK1 and MNK2 enforce expression of E2F1, FOXM1, and WEE1 to drive soft tissue sarcoma (Ke et al., 2021). In other types of cancer, like in hepatocellular carcinoma, scientists have proved that long non-coding RNA CDKN2B-AS1 could enhance tumor progression via the E2F1/G protein subunit alpha *Z*-axis (Tao et al., 2023). Targeting the E2F1/Rb/HDAC1 axis with the small molecule HR488B effectively inhibits colorectal cancer growth (Duan et al., 2023). Besides, CYCLINB1 protein had the most significant relation with TUBB mRNA expressions. CYCLINB1 has also been well-studied in multiple cancers. For example, Lv S et al. found that inhibiting CYCLINB1 resulted in suppressed proliferation, invasion, and epithelial mesenchymal transition of hepatocellular carcinoma cells and enhanced sensitivity to TRAIL-Induced apoptosis (Lv et al., 2020). Also, CYCLINB1 is a defined biomarker in esophageal squamous cell carcinoma (Li et al., 2023), penile squamous cell carcinoma (Tan et al., 2022), gastric cancer (Li et al., 2021), breast cancer (Liu et al., 2019), nasopharyngeal carcinoma (Xie et al., 2019), *etc.* As an important factor in so many cancers, our studies provided a new idea that TUBB may function in tumor formation by interacting with the CYCLINB1 protein.

In this study, we found that TUBB was generally differentially expressed between tumor tissues and normal tissues through a full-scale pan-cancer analysis, using a series of bioinformatics approaches. Correlation between TUBB expressions and clinical prognosis was uncovered in the heatmap, forest plots, and ROC curves, making our findings more reliable. TUBB was demonstrated as a potential independently working prognostic factor for most tumors. In particular, TUBB’s risky role was emphasized. We confirmed that TUBB may be the reason why immune rejection and immune escape should be generated. Higher expressions of TUBB would result in less immune-related molecules in pan-cancer. Aiming at this, we further conducted drug resistance analysis and found that TUBB played an important role in regulating chemotherapy sensitivity across cancers. But this was controversial, reminding us to perform more and better research and development of drugs with safer and more effective clinical trials. At last, we selected SARC to study more. And we offered E2F1 as a key gene interacting with TUBB. Moreover, CYCLINB1 was identified as another hub term in TUBB expressions. These results mutually provided novel insights in future studies on TUBB in cancers, and they are hoped to be helpful to further elucidate the explicit mechanisms of TUBB in cancer initiation and progression.

In a nutshell, using the multi-omics method, TUBB has been elucidated to be dysregulated in pan-cancers as a potential diagnostic marker. The article systematically describes the relationship between TUBB and clinical outcomes in pan-cancers. For the first time, the possibility of targeting TUBB with drugs and small molecules has been identified from multiple dimensions. Moreover, a large number of single-cell datasets were combined to identify cells expressing TUBB at high resolution. The pathway and metabolic disorders mediated by TUBB were validated, and the transcription factors regulating their expressions were identified using the CHIP-seq approach. TUBB expression was linked to multiple molecular subtypes, demonstrating the potential of stratified precision therapies.

## Figures and Tables

**Table:** 

**TME**	tumor microenvironment
**TCGA**	the Cancer Genome Atlas
**TUBB**	β-tubulin
**PTMs**	post-translational modifications
**TIME**	tumor immune microenvironment
**GTEx**	the Genotype-Tissue Expression project
**AUC**	area under curve
**IHC**	immunohistochemical
**OS**	overall survival
**DSS**	disease-specific survival
**PFI**	progression free interval
**DFI**	disease free interval
**KM**	Kaplan-Meier
**SCNA**	Somatic copy number alteration
**CNAs**	copy-number alterations
**GSEA**	Gene Set Enrichment Analysis
**SARC**	sarcoma
**TME**	tumor microenvironment
**GSVA**	Gene Set Variation Analysis
**NCI**	National Cancer Institute
**HR**	Hazard ratio
**CI**	confidence interval
**THYM**	thymoma
**IC50**	half maximal inhibitory concentration
**XSum**	Extreme Summarization
**MTAs**	microtubule-targeting agents
**MSAs**	microtubule-stabilizing agents
**MDAs**	microtubule-destabilizing agents
**NSCLC**	non-small cell lung cancer
**miRNAs**	microRNAs
**IFN-γ**	interferon-gamma
**CTL**	cytotoxic T lymphocyte

## Data Availability

The datasets presented in this study can be found in online repositories. The names of the repository/repositories and accession number(s) can be found in the article/Supplementary Material.

## Supplementary material

SUPPLEMENTARY FIGURE S1 TUBB mRNA levels between tumor and normal tissues using GEO data from different cancers.

SUPPLEMENTARY FIGURE S2 Differences in TUBB expressions in different tumor stages by Kruskal-Wallis Rank Sum test in ACC, ESCA, KIRC, KIRP, LIHC, SKCM, and STAD.

SUPPLEMENTARY FIGURE S3 ROC curves (AUC value > 0.7) demonstrated the diagnostic value of TUBB in distinguishing tumors based on the TCGA data.

SUPPLEMENTARY FIGURE S4 ROC curves (AUC value > 0.7) demonstrated the diagnostic value of TUBB in distinguishing tumors based on the TCGA-GTEx data.

SUPPLEMENTARY FIGURE S5 The difference of TIP scores between high and low expression groups of TUBB was calculated.
