# Supplementary material for: TUBB, a robust biomarker with satisfying abilities in diagnosis, prognosis, and immune regulation via a comprehensive pan-cancer analysis
Source: Front Mol Biosci. 2024 May 2;11:1365655. doi: 10.3389/fmolb.2024.1365655 (PMC11096532; doi:10.3389/fmolb.2024.1365655)

Supplementary Material

Title: TUBB, a robust biomarker with satisfying abilities in diagnosis, prognosis, and immune regulation via a comprehensive pan-cancer analysis

Zaifu Zhu[a†], Wei Zhang[b†], Shaohu Huo[a], Tiantuo Huang[a], Xi Cao[cd*], Ying Zhang[ef*]

a Department of Pediatrics, First Affiliated Hospital of Anhui Medical University, Hefei, Anhui Province, China.

b Department of Rehabilitation Medicine, First Affiliated Hospital of Anhui Medical University, Hefei, Anhui Province, China.

c Department of Pharmacy, First Affiliated Hospital of Anhui Medical University, Hefei, Anhui Province, China.

d The Grade 3 Pharmaceutical Chemistry Laboratory of State Administration of Traditional Chinese Medicine, Hefei, Anhui Province, China

e Department of Pathology, First Affiliated Hospital of Anhui Medical University, Hefei, Anhui Province, China.

f Pathology Center, Anhui Medical University, Hefei, Anhui Province, China.

† These authors have contributed equally to this work and share first authorship.

*Corresponding authors:

Ying Zhang, Department of Pathology, First Affiliated Hospital of Anhui Medical University, Hefei, Anhui Province, China. Email: zy1122812@163.com.

Xi Cao, Department of Pharmacy, First Affiliated Hospital of Anhui Medical University, Hefei, Anhui Province, China. Email: caoxi@ahmu.edu.cn.

Supplementary Fig.1 TUBB mRNA levels between tumor and normal tissues using GEO data from different cancers.

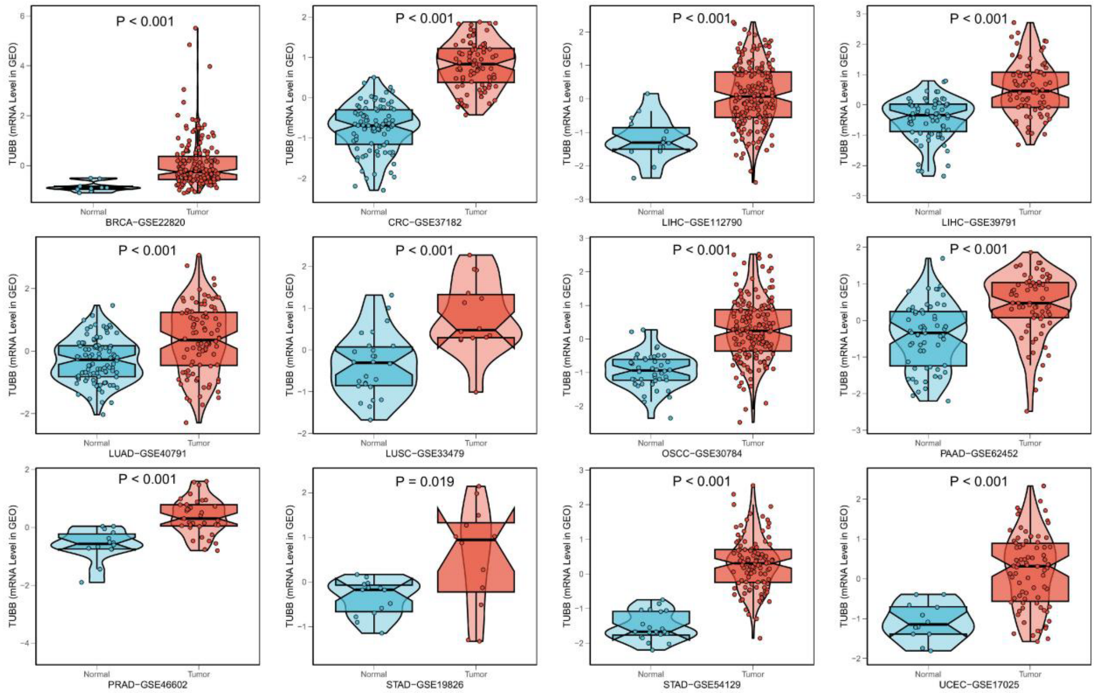

Supplementary Fig.2 Differences in TUBB expressions in different tumor stages by Kruskal-Wallis Rank Sum test in ACC, ESCA, KIRC, KIRP, LIHC, SKCM, and STAD.

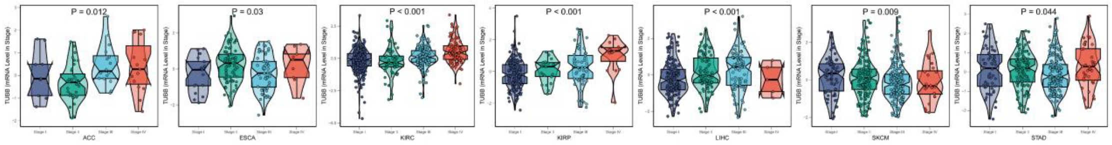

Supplementary Fig.3 ROC curves (AUC value>0.7) demonstrated the diagnostic value of TUBB in distin guishing tumors based on the TCGA data.

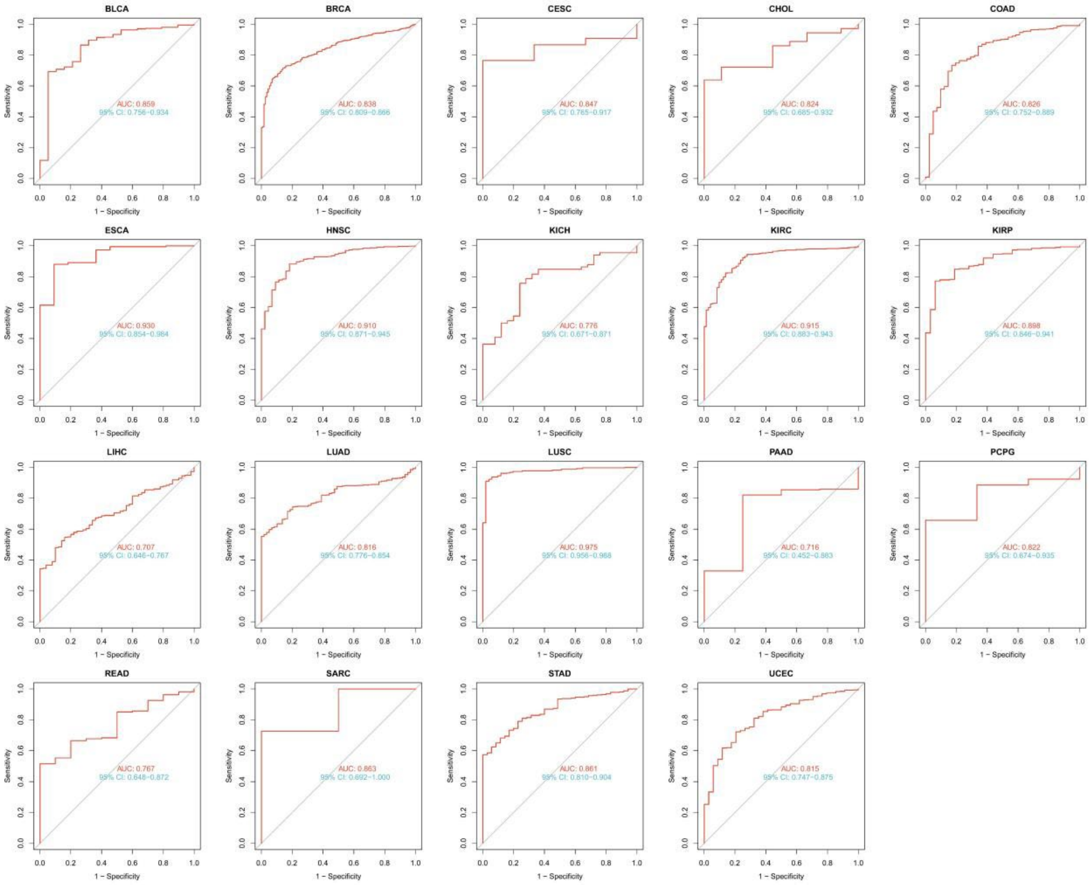

Supplementary Fig.4 ROC curves (AUC value>0.7) demonstrated the diagnostic value of TUBB in distinguishing tumors based on the TCGA-GTEx data.

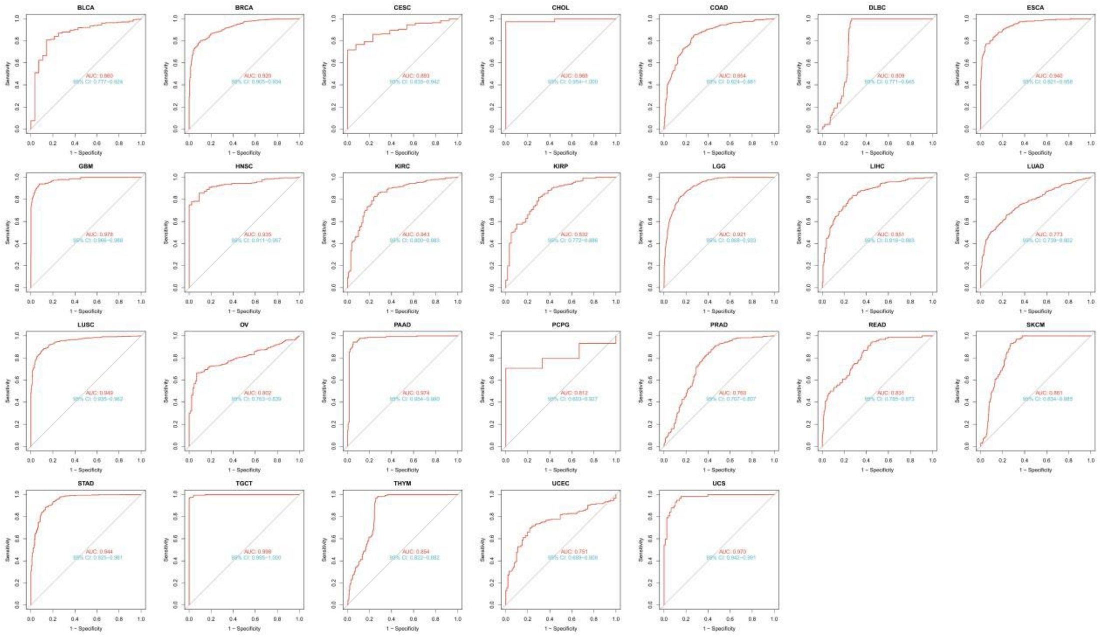

Supplementary Fig.5 The difference of TIP scores between high and low expression groups of TUBB w as calculated.

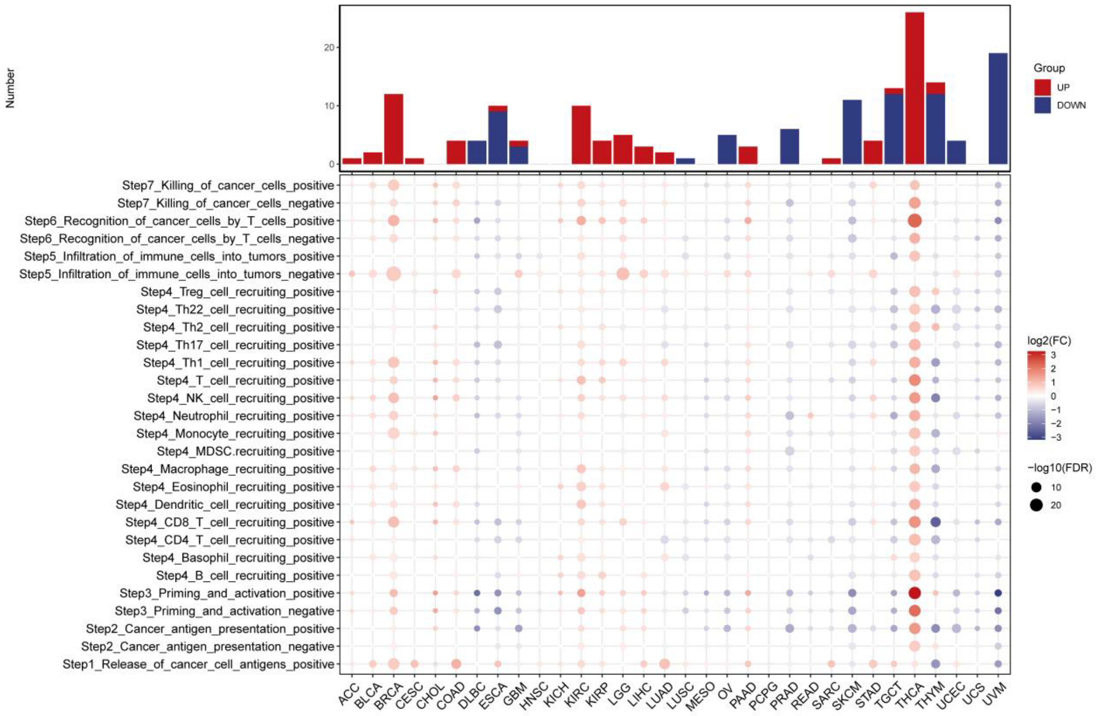

Supplement: Supplementary file 1 [file Image1.pdf]
